# Complete genome sequence of *Thermaerobacter composti* strain Ins1, a spore-forming filamentous bacterium isolated from a deep geothermal reservoir

**DOI:** 10.1128/mra.00058-24

**Published:** 2024-03-13

**Authors:** Danae Bregnard, Diego Gonzalez, Eva Di Francesco, Naïma Mangia, Simona Regenspurg, Pilar Junier

**Affiliations:** 1 Laboratory of Microbiology, University of Neuchâtel, Neuchâtel, Switzerland; 2 Geoenergy, GFZ German Research Centre for Geosciences, Potsdam, Germany; California State University San Marcos, San Marcos, California, USA

**Keywords:** complete genome, geothermal energy, bacteria

## Abstract

We report the complete genome sequence of *Thermaerobacter composti* strain Ins1, a gram-positive filamentous spore-forming bacterium, isolated from deep geothermal fluids used for electricity production. This is the first complete (circular) genome assigned to the species *Thermaerobacter composti*.

## ANNOUNCEMENT

Members of the *Thermaerobacter* genus are thermophilic bacteria isolated from high-temperature environments including compost (*Thermaerobacter composti* JCM 15650 strain Ni80^T^ (1), the Mariana Trench (*Thermaerobacter marianensis* 7p75a^T^) (2), shallow hydrothermal vents (*Thermaerobacter nagasakiensis* Ts1a^T^) (3), a bore run-off channel (*Thermaerobacter subterraneus* C21^T^) (4), and a hydrothermal beach (*Thermaerobacter litoralis* KW1^T^) (5). Strain Ins1 was isolated from fluids of the production well of the deep geothermal reservoir (163°C; 3,650 m depth) of an electricity production plant in the Upper Rhine Graben (49.1571411 N, 8.1480501 E). After enrichment in Marine Broth 2216 (BD Difco, USA) at 60°C without agitation, only one bacterium grew upon isolation on solid Marine Broth 2216 (1.5% agar).

Genomic DNA was extracted from a 10-day-old culture using the Wizard HMW DNA Extraction Kit (Promega, USA). Sequencing was performed on a Pacific Biosciences Sequel II instrument after DNA shearing, size selection, and HiFi SMRTbell Library construction (chemistry version: 11.1.0.154383; average insert size: 7,718 bp). A genome was assembled from 34,272 HiFi reads using *flye* (v. 2.9.2) (6) with 2,000 bp minimum overlap and three polishing rounds (average coverage 89×); the chromosome was circularized by *flye* based on the assembly graph. One consistently methylated motif (GC^m6^ANNNNNNGTT/A^m6^ACNNNNNNTGC, >99% detection rate) was identified by *Smrtlink* (v. 11). Assembly quality was assessed using *CheckM* (estimated completeness: 97.03%; contamination rate: 0.53%). The circular assembled genome corresponded to 3,001,239 bp with a 73% G + C content.

The genome was annotated using NCBI Prokaryotic Genome Annotation Pipeline (PGAP v.6.5). It included 2,363 protein-coding genes, 2 copies of each ribosomal RNA gene, 47 tRNAs, and 4 non-coding RNAs (signal recognition particle sRNA, 6S RNA, RNA component of RNase P, and the transfer-messenger RNA); 54 additional putative protein-coding genes were predicted to be non-functional (pseudogenes).

Strain Ins1 was assigned to the *Thermaerobacter composti* species. The 16S rRNA gene was 99.94% identical with the sequence of *T. composti* JCM 15650 based on pairwise alignment (NCBI *blast*). Digital DNA-DNA hybridization (https://ggdc.dsmz.de, dDDH, formula 2) was 86.2%, which falls within the limits of the same bacterial species (7). A phylogenetic tree based on 28 concatenated ribosomal/conserved proteins (elongation factor G/elongation factor Tu/‍L14/‍L15/‍L16/‍L17/‍L18/‍L2/‍L22/‍L23/‍L24/L29/L3‍/L4‍/L5‍/L6‍/S10‍/S11‍/S12‍/S13‍/S14‍/S17‍/S19‍/S3‍/S4‍/S5‍/S7‍/S8‍) aligned using *muscle* (v. 3.8.31) (8) was constructed by *iqtree* (v. 2.2.5) (9) with automated model selection, confirming that strain Ins1 clustered within the *Thermaerobacter* genus, closest to strain FW80 (10).

The amino acid composition of the full proteome showed a limited ERK bias around −15 (11), suggesting that the strain is moderately thermophilic [50°C–60°C predicted optimal growth temperature; 3.08 hours doubling time based on ribosomal protein genes, by *gRodon* v. 2.3.0 (12)]. Presence in geothermal water is likely due to the production of heat-resistant endospores. Accordingly, the genome encodes 58 out of 66 proteins considered essential for sporulation in Clostridiales (13), and endospores were observed in old cultures (1). Metabolic pathway analysis using *gapseq* v. 1.2 (14) suggested that the strain requires exogenous thiamine and may be auxotrophic for several amino acids (Try, His, Ser, Phe, Pro, Thr, Tyr, Val).

## Data Availability

This whole-genome shotgun project has been deposited at NCBI under the accession CP132508; SRAs are available under accession SRR27458461.
